# Evaluation of chlorophyll-loaded mesoporous silica nanoparticles for photodynamic therapy on cancer cell lines

**DOI:** 10.1007/s10103-024-03988-2

**Published:** 2024-01-23

**Authors:** Fadya Adnane, Soliman Mehawed Abdellatif Soliman, Emad ElZayat, Essam M. Abdelsalam, Heba Mohamed Fahmy

**Affiliations:** 1 Biotechnology Department, Faculty of Science, Cairo University, Cairo, Egypt; 2 Chemistry Department, Faculty of Science, Cairo University, Cairo, Egypt; 3 Laser Applications in Metrology, Photochemistry, and Agriculture (LAMPA) Department, National Institute of Laser Enhanced Sciences (NILES), Cairo University, Cairo, Egypt; 4 Biophysics Department, Faculty of Science, Cairo University, Cairo, Egypt

**Keywords:** Photodynamic therapy, Mesoporous silica nanoparticles (MSNs), Chlorophyll (Chl), HepG2, MDA-MB-231, HSF

## Abstract

Chlorophyll (Chl) is a promising natural photosensitizer (PS) in photodynamic treatment (PDT). Mesoporous silica nanoparticles (MSNs) were chosen to increase the effectiveness of PDT. This study aimed to evaluate the synergistic efficacy of chlorophyll-loaded mesoporous silica nanoparticles (Chl-MSNs) with photodynamic therapy (PDT) and to investigate their potential toxicity in HepG2, MDA-MB-231, and HSF cell lines. Chl-MSNs were prepared via the physical adsorption method. TEM, DLS, and zeta potential examined morphology, size, and surface characteristics. MSNs and Chl-MSNs were characterized using the same techniques. HPLC was used to assess the encapsulation efficiency. At pH 7.4, an in vitro release experiment of Chl-MSNs was performed. Chl, MSNs, and Chl-MSNs were applied to the three cell lines at different concentrations and subjected to red (650 nm) and blue (450–500 nm) lasers. MSNs and Chl-MSNs’ sizes were 90.338 ± 38.49 nm and 123.84 ± 15.67 nm, respectively, as obtained by TEM; the hydrodynamic diameter for MSNs (93.69 ± 20.53 nm) and Chl-MSNs (212.95 ± 19.76 nm); and their zeta potential values are − 16.7 ± 2.19 mV and − 18.84 ± 1.40 mV. The encapsulation efficiency of Chl-MSNs was 70%. Chl-MSNs displayed no toxicity in dark conditions but showed excellent photostability under blue and red light exposure. Furthermore, using Chl over Chl-MSNs has a higher PDT efficiency than the tested cell lines. Chl-MSNs have the potential to be an effective delivery system. PDT proved to be an essential technique for cancer treatment. Blue laser is recommended over red laser with Chl and MSNs for destroying cancer cells.

**Graphical Abstract:**

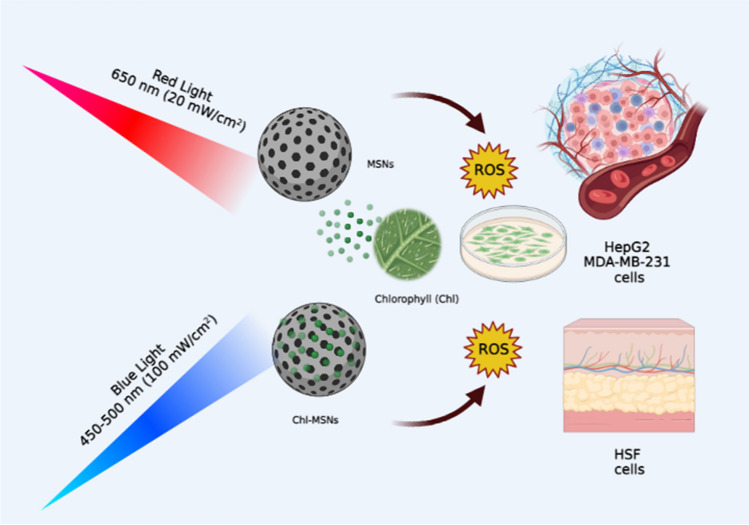

## Introduction

Photodynamic therapy (PDT) is an alternative to chemotherapy and radiation therapy for treating and inhibiting the spread of malignant tumor cells [1]. Three essential elements are needed to apply this strategy correctly: a photosensitizer (PS), tissue oxygen, and a source of light energy [2, 3]. The PS agent is consequently localized to the targeted cell and activated using light energy. Light stimulation produces a significant amount of reactive oxygen species (ROS), significantly increasing the targeted cells’ cytotoxicity [4]. Furthermore, the targeted tumor cells’ vascular structure is damaged by ROS, which triggers the cells’ inflammatory response, resulting in apoptosis [5–8]. Researchers have attempted to develop new natural photosensitizers that can be excited within the range of 600–850 nm, which is called “the phototherapeutic window” and acts as an optimum range for tissue permeability, which leads to the generation of an intense electronic transition in the phototherapeutic window and further improves light penetration [9]. Therefore, chlorophyll (Chl) will act as the most suitable light-sensitive pigmented substance or photosensitizer in absorbing photons and releasing electrons [10].

Chlorophyll is found in green plants as two main chemical structures, Chl a and Chl b, typically in a 3:1.1 ratio [11]. Chlorophyll a maximally absorbs within the red light regions at 642 nm and in the orange light region absorbs at 372 nm. For the blue region, on the other hand, chlorophyll b has maximal absorption at 626 nm and 392 nm in the red and blue light regions, respectively, which makes chlorophyll a perfect choice as a photosensitizer [12]. Chlorophyll has proved to be an effective bioactive chemopreventive agent because it can generate promising effects toward mutagens and carcinogens and limit cancer development [13, 14]. Chlorophylls have excreted multiple biological activities as anticancer agents like antigenotoxicity [15], trapping of mutagens [16], antioxidant activities, apoptosis, and immunomodulation [17]. Chlorophyll has some limitations associated with being in its pure natural form; its weak stabilization under physiological environments because the hydrophobic porphyrin aromatic ring forms chlorophyll accumulations, giving an inefficient biological sensitizing action and poor solubility in aqueous solutions, decreasing its accumulation in cancer cells [10].

Mesoporous silica nanoparticles (MSNs) are ideal nanosystems for loading therapeutic biomolecules due to their extended surface area and numerous pores [18]. They have a mild pH response, are quickly destroyed in nature, and have less toxic effects [19, 20]. Therefore, MSNs are the most valuable and suitable nanoparticles for delivering and carrying a variety of chemical compounds, such as drugs and antioxidants [21–23]. The interaction between nanoparticles and chlorophyll has a significant role in improving the photo-chemo properties of chlorophylls, especially in mesoporous silica nanoparticles, as this conjugation gained higher stability in the aqueous environment and higher stability against light radiation, which exerts a higher photosensibility action in the long duration of light exposure [24]. The present work aims to show the synergistic effects of photodynamic therapy with nanotechnology and to improve the capability of PDT in destroying cancer cells by exploring the possible toxicity of chlorophyll-loaded mesoporous silica nanoparticles (Chl-MSNs) with blue and red light irradiation in HepG2, MDA-MB-231, and HSF cell lines.

## Materials and methods

### Materials

TEOS 99%, CTAB 99%, DMSO, ethanol 99%, and CDNB 30 mmol/L were bought from Sigma-Aldrich in St. Louis, MO, USA. NH4OH, 28%, was obtained from Fluka. Sigma-Aldrich (Germany) provided trypsin, FBS 10%, DMEM/F12 medium, L-glutamine, penicillin, MTT, streptomycin, DMSO, PBS, and 70% (v/v) ethanol from Thermo Fisher Scientific Inc. (Waltham, MA, USA). Chlorophyll was bought from Unicity Health Private Ltd. (India) as a super chlorophyll dietary supplement powder.

### Methods

#### Mesoporous silica nanoparticle (MSN) synthesis

Elbialy et al. used a technique to prepare MSNs, which involved dissolved CTAB in deionized water, combined with 2-ethoxyethanol and 28% NH_4_OH, stirred for 30 min, added TEOS, centrifuged for 15 min, washed three times with ethanol, deionized water, and dried for 6 h at 500 °C to remove CTAB [25].

#### Chlorophyll-loaded mesoporous silica nanoparticles (Chl-MSNs) preparation

The loading method was performed by [24, 26] by only the concentration of 2.0 × 10 − 4 M, which was the highest concentration. Chl-MSNs were prepared via physical adsorption by adding an equal ratio of MSNs in an ethanolic solution containing Chl. The suspension was then shaken for 30 min in the shaker at 25 °C until equilibrium was established. Subsequently, to measure the concentration of free Chl, the Chl-MSN solution was centrifuged, and the supernatant was collected, which was determined from a calibration curve with 253 nm a spectrophotometer (Jenway UV-6420; Barloworld Scientific, Essex, UK). HPLC was also used to measure the Chl concentration (the free drug). Using the following equation, the encapsulation efficiency can be calculated:

$$\mathrm E\mathrm E\%=(\frac{(Total\;drug-Free\;drug)}{Total\;drug})x100$$

#### Nanoformulations’ physical characterization

##### Transmission electron microscope (TEM)

The morphological information of MSNs and Chl-MSNs was examined using TEM (JEM 1230 electron microscope Jeol, Tokyo, Japan), and the nanoparticle mixtures were filtered and dried before testing on a carbon grid coated with copper.

##### Particle size and zeta potential assessments using dynamic light scattering (DLS)

DLS was used to measure the particle size distribution of MSNs and Chl-MSNs, evaluating sample quality by providing information about the polydispersity indexes (PDI) of the nanoparticles. The hydrodynamic diameter was established, and the size distribution and surface charge were investigated using a zeta sizer (Nano ZS, Malvern Instruments, Malvern, UK). Each measurement’s mean values and standard errors (S.E.M.) were determined after using triplicate values.

## In vitro drug release study

Mohseni et al. described a method for measuring the in vitro release of Chl from Chl-MSNs using a dialysis bag [27]. Chl-MSNs were soaked in a pH 7.4 PBS solution, centrifuged, and redispersed in PBS. Bottles were filled with release media, shaken, and re-suspended at different intervals of (0.5, 1, 2, 3, 4, 5, and 24 h). Chl concentrations were determined using a UV–Vis spectrophotometer at 405 nm.

### Cell culture treatment

HepG2, MDA-MB-231, and HSF cells were cultured in a DMEM medium provided with penicillin (100 U/mL), 10% FBS, streptomycin (100 mg/L), and L-glutamine (2 mM). After treatment, cells were sown in 96-well plates, adhering for 24 h to 70% confluence. Non-attached cells were discarded.

### Photodynamic therapy treatment

Chl, MSNs, and Chl-MSNs were dissolved in 1 mL DMSO (100%). The cells were treated with Chl, MSNs, and Chl-MSNs at different concentrations (400, 200, 100, 50, 25, and 12.5 μg/mL); there were two types of controls: untreated cells without irradiation (negative control) and untreated cells with laser. After 48 h incubation, the culture plate was irradiated with a diode laser (CivilLaser (CL), NaKu Technology Co., Ltd., Zhejiang, China) at an excitation wavelength of 652 nm for red laser, 2–4 W average power, with a light intensity of (20 mW/cm^2^) and energy of 12.10 J. For the blue laser, the excitation wavelength was 450–500 nm, with a light intensity of (100 mW/cm^2^) and energy of 60.00 J. The plates were irradiated with blue or red laser over a specific time of 600 s. The distance from the light source to the surface of the plates was adjusted to be about 10 cm. The irradiation was carried out in quadruplicate for each concentration. The MTT viability assay was performed after treatment.

### MTT viability assay

The MTT assay was used to estimate cell proliferation of HepG2, MDA-MB-231, and HSF cell lines. Culture plates were washed and incubated with 0.5% MTT reagent, and optical densities (OD) were measured using an ELISA reader Biotek 8000; USA) at 570 nm (DMSO) and 492 nm (SDS) [28]. The following equation was used to estimate the cell viability percentage [29]:

$$\mathrm{Viability}\;\mathrm{percentage}\;(\%)=\frac{\mathrm{OD}\;\mathrm{of}\;\mathrm{treated}\;\mathrm{cells}}{\mathrm{OD}\;\mathrm{of}\;\mathrm{untreated}\;\mathrm{cells}}x100.$$

### Statistical analysis

The data were expressed as the mean of triplicates ± standard errors (S.E.M.) for the physical characterization, including TEM, DLS, and zeta potential measurements of the nanoformulations and four replicates ± standard deviation (SD) for the cytotoxicity MTT assay, which was then analyzed using GraphPad Prism 7.00.

## Results

### Physical characterization of MSNs and Chl-MSNs

**Fig. 1:**
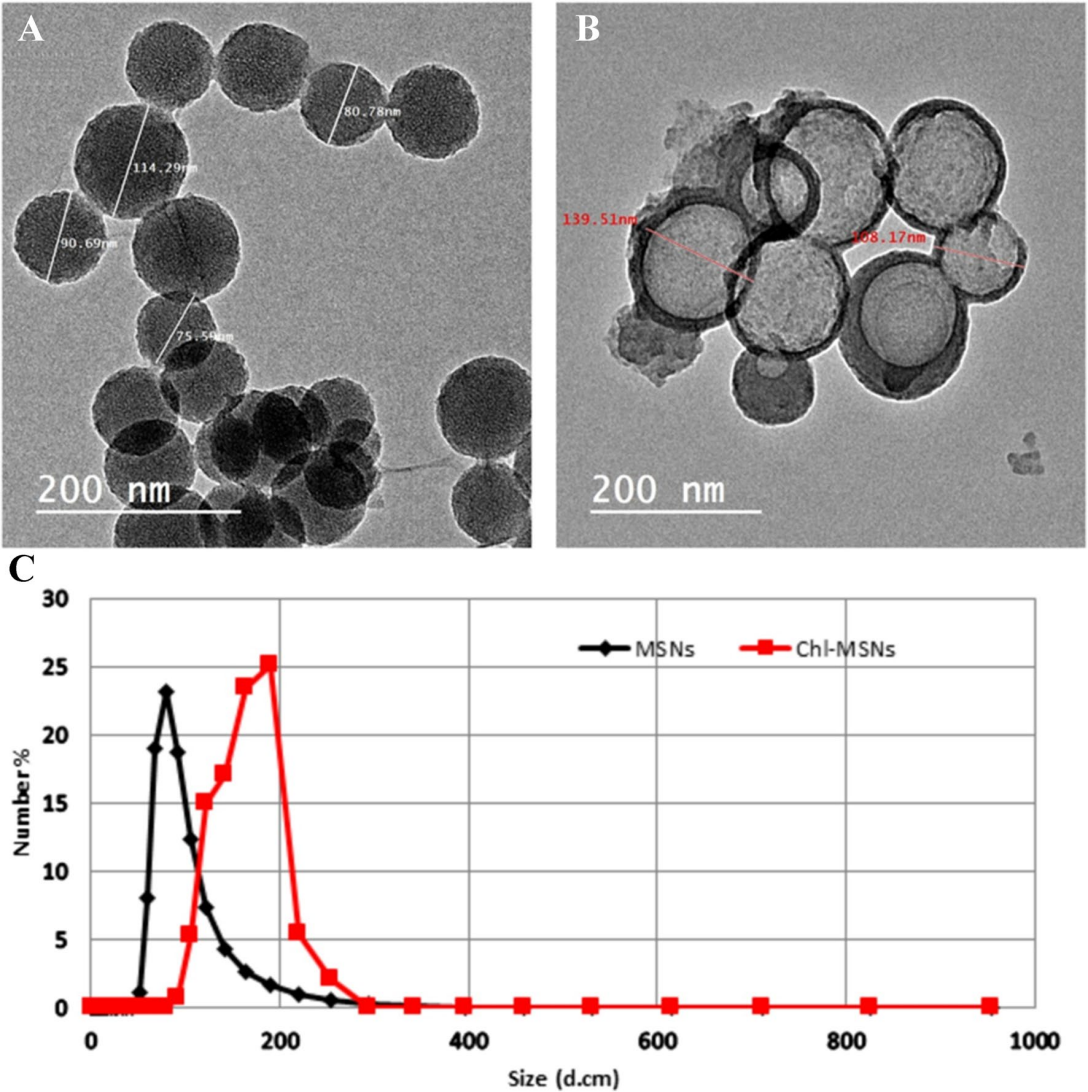
**A** MSN mesoporous silica nanoparticle TEM micrograph (90.338 ± 38.49 nm). **B** Mesoporous silica nanoparticles loaded with chlorophyll Chl-MSN TEM micrograph (123.84 ± 15.67 nm). **C** Particle size distribution of MSNs (93.69 ± 20.53 nm) and Chl-MSNs (212.95 ± 19.76 nm)

The surface morphology of MSNs and Chl-MSNs was investigated using TEM and showed a spherical homogeneous size distribution of about 90.338 ± 38.49 nm for MSNs and 123.84 ± 15.67 nm for Chl-MSNs (Fig. 1A and 1B). The TEM micrograph also shows a symmetrical structure with regular pore alignment and negligible aggregation. The results of DLS analyses revealed that MSNs and Chl-MSNs had mean hydrodynamic diameters of 93.69 ± 20.53 nm and 212.95 ± 19.76 nm, respectively, which reveals that the average particle size of Chl-MSNs is slightly larger than MSNs (Fig. 1C). Additionally, MSNs and Chl-MSNs had PDI values of 0.424 and 0.41, respectively. According to the zeta potential measurements, both MSNs and Chl-MSNs have net surface negative charges with average values of − 16.7 ± 2.19 mV and − 18.84 ± 1.40 mV, respectively. The encapsulation efficiency of Chl-MSNs was found to be 70% with a weight ratio of 1:1 for MSNs: Chl, which can be attributed to the large surface pores of MSNs that can hold significant amounts of drugs and the potent electrostatic interaction between the negative charge in MSNs and the positive charge in Chl.

### In vitro release kinetics of Chl from Chl-MSNs

**Fig. 2:**
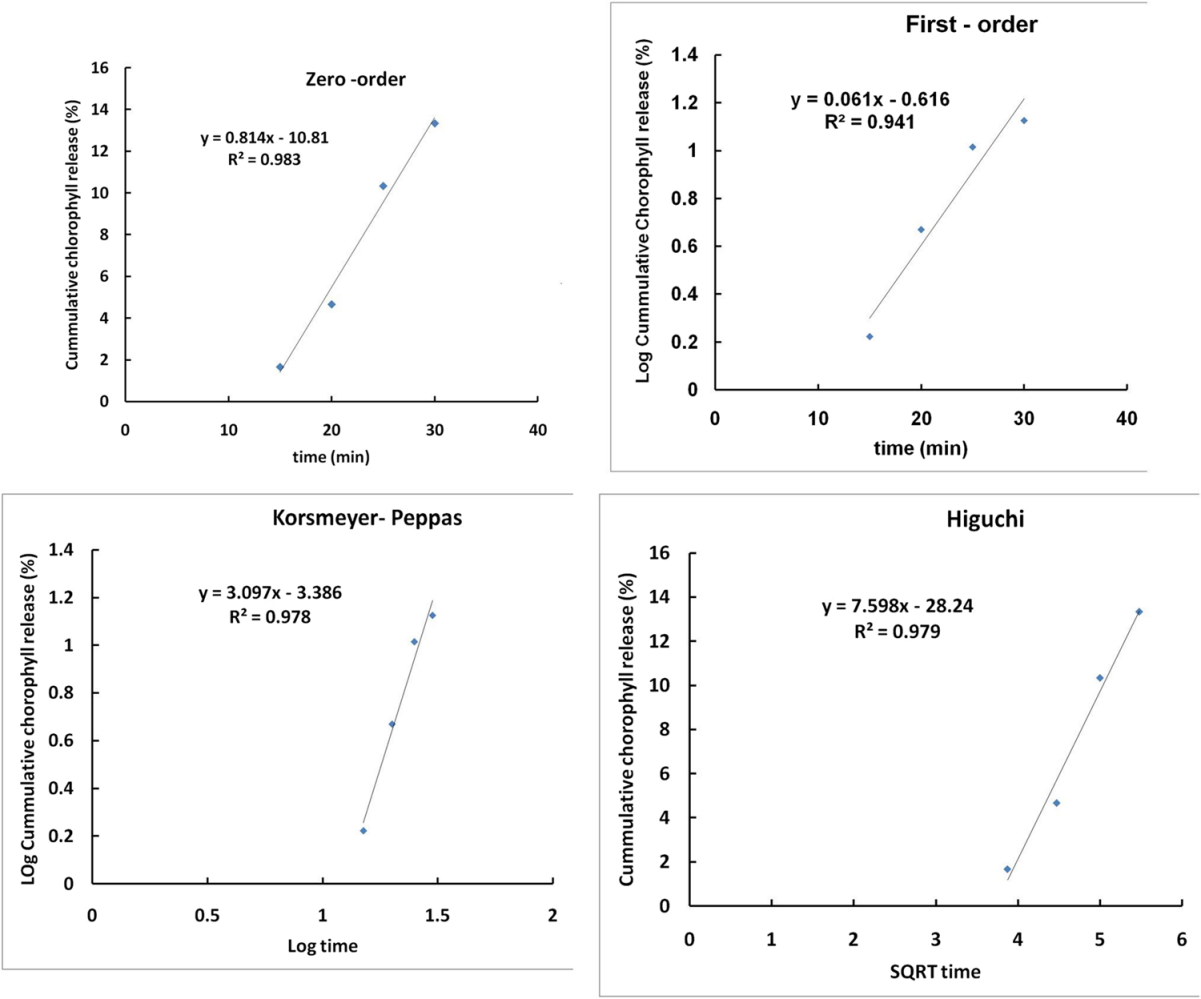
Kinetic analyses of vitro release pattern of Chlorophyll (Chl) from Chl-loaded MSNs (MSNs): zero order, first order, Korsmeyer-Pappas, and Higuchi models

Kinetic parameters of chlorophyll release are shown in Fig. 2 and Table 1. The behavior of the release was studied based on mathematical models such as the zero order, first order, Korsmeyer-Peppas, and Higuchi, according to Eqs. (1)–(4). Zero-order, first-order, and Higuchi relations with correlation coefficients (R2) were 0.98, 0.94, and 0.98, respectively, indicating the controlled release of chlorophyll with independent chlorophyll concentration. Zero order and first order refer to the slow release of chlorophyll into solution in the same amount per unit of time. The Higuchi relation represents the mechanism of releasing chlorophyll from nanoparticles into a solution through diffusion. According to the Korsmeyer-Pappas model, as shown in the figure, the relation with correlation coefficients (*R*2) equal 0.98, and the “*n*” value is higher than one that indicates the mechanism of transportation of chlorophyll is super case II transport [30]. As a result, a rapid release was detected during the first 30 min of the Chl from Chl-MSNs’ in the in vitro release experiment. However, the release of Chl from the MSNs’ inner porosities most likely caused the delayed release at a later time.

**Table 1:** Study the kinetics of chlorophyll (Chl) release using mathematical models

Kinetic models	Parameters	Value
Zero order	*R*^2^	**0.98**
First order	*R*^2^	**0.94**
Korsmeyer-Peppas	*R*^2^	**0.98**
	*n*	**3.09**
Higuchi	*R*^2^	**0.98**

The equations of mathematical models [31]:

$$\mathrm{Zero}\;\mathrm{order}\;\mathrm{model}\;Q=K_0\;t$$

$$\mathrm{First}\;\mathrm{order}\;\mathrm{model}\;Q_t=Q_0e^{kt}$$

$$\mathrm{Higuchi}\;\mathrm{kinetic}\;\mathrm{model}\;Q=K_H\sqrt t$$

$$\mathrm{Korsmeyer}-\mathrm{Pappas}\;\mathrm{model}\frac M{M_\infty}=Kt^n$$

*Q* is the amount of chlorophyll at time *t*, *Q*_o_ is the amount of chlorophyll initially in nanoparticles, *t* is the time, KH is the Higuchi constant, and *K*_o_ and *K* are the zero-order and first-order release constants, respectively.

### MTT cell cytotoxicity test

The cytotoxicity test was investigated using the MTT assay against HepG2, MDA-MB-231, and HSF cell lines. The data are presented as IC50 according to the dose-dependent cytotoxicity that Chl, MSNs, and Chl-MSNs have shown under dark and PDT conditions. In the dark cytotoxicity test, the IC50 values of Chl, MSNs, and Chl-MSNs for HepG2, MDA-MB-231, and HSF cells were > 400 μg/mL. Under red light exposure, for HepG2 cells, the IC50 values of Chl, MSNs, and Chl-MSNs were 129.0 μg/mL, 65.59 μg/mL, and 143.9 μg/mL, respectively, and under blue light radiation, the values were 37.43 μg/mL, 14.44 μg/mL, and 310.9 μg/mL, respectively. For MDA-MB-231, in red light exposure with Chl, MSNs, and Chl-MSNs, the IC50 values were all > 400 μg/mL. In the blue laser, the IC50 values were 18.89 μg/mL, 143.6 μg/mL, and 108.3 μg/mL, respectively. The IC50 values of red light application on normal HSF cells with Chl, MSNs, and Chl-MSNs were showed to be 0.359 μg/mL, 1.173 μg/mL, and 0.3226 μg/mL, respectively, and for blue laser, the values were 3.078 μg/mL, 31.17 μg/mL, and 63.71 μg/mL, respectively (Table 2).

**Table 2:** IC50 values of Chl, MSN, and Chl-MSN cytotoxicity against HepG2, MDA-MB-231, and HSF cell lines after exposure to light intensities of blue (100 mW/cm2) and red (20 mW/cm2) lasers for 600 s. The studies were carried out twice in quadruplets, with concentrations in μg/mL

Cell line type	Blue laser			Red laser		
	Chl	MSNs	Chl-MSNs	Chl	MSNs	Chl-MSNs
HepG2	37.43	14.44	310.9	129.0	65.59	143.9
MDA-MB-231	18.89	143.6	108.3	> 400	> 400	> 400
HSF	3.078	31.17	63.71	0.359	1.173	0.3226

## Discussion

MSNs have garnered much attention as potential inorganic nanocarriers because of their high porosity and simplicity in surface modification, and they have several benefits over organic nanocarriers, including rigid structure, mechanical, chemical, and thermal stability, controlled release, and high loading efficiency [32, 33].

DLS measurements provided by the mean of the hydrodynamic diameter showed that the size of particles of Chl-MSNs (212.95 ± 19.76 nm) was more significant than the free MSNs (93.69 ± 20.53 nm). This enlargement could be explained by Chl adhesion to the pores of the MSNs. TEM confirmed this increase in size, showing a size distribution of 90.338 ± 38.49 nm for MSNs and 123.84 ± 15.67 nm for Chl-MSNs. The PDI values measure the homogeneity and uniformity of the particle size distribution. PDI values indicate a narrow size distribution between 0.1 and 0.5, whereas a broad distribution is indicated by PDI values greater than 0.5. In this study, the PDIs of MSNs and Chl-MSNs were 0.424 and 0.41, respectively, indicating that the synthesized preparation has a homogenous distribution. It is generally recognized that low PDI values are required for drug delivery systems to enhance pharmacokinetic characteristics like distribution and absorbance [34].

Zeta potential evaluation is a helpful tool for determining particles’ surface charges. This parameter displays the extent to which the charged particles in the dispersion repel one another. The colloidal system’s potential stability is shown by the zeta potential value, as the suspension with particles with a high negative or positive zeta potential tends to repel each other and resist aggregation. For low zeta potential values, particles attract, and the mixture is likely to coagulate [35]. Chl-MSNs have a sufficiently negative zeta potential charge (− 18.84 1.40 mV) to maintain their stability for a considerable time. Furthermore, the modification of MSNs is ensured by the difference in the zeta potential due to conjugation with Chl [36]. The encapsulation efficiency of Chl-MSNs was found to be 70% because MSN’s pores have an enormous surface area and can retain many drugs, as well as the strong electrostatic interaction between the negative charge in MSNs and the positive charge in Chl, indicating that the mechanism of chlorophyll transportation is super case II transport [30]. Chl from Chl-MSNs was released quickly over 30 min, according to the in vitro release study. However, the release of Chl from the internal pores of MSNs most likely contributed to the later, slower release. In this study, Chl was loaded in MSNs using the physical adsorption method, which may be classified as a monophasic drug delivery system because most of Chl was released within 0.5 h.

Chl was applied to HepG2 cells in the dark at six concentrations comparable to the same concentrations of MSNs and Chl-MSNs prepared. The same procedure was applied to the MDA-MB-231 and HSF cell lines. The research showed that high concentrations of Chl had a noticeable inhibitory effect, which was observed in the three cell lines. The inhibition of xenobiotic metabolizing enzymes, activation of apoptosis in cancer cell lines, and antioxidant and antimutagenic activity contribute to cancer prevention [17]. This is similar to the findings of other research, which discovered that Chl limits the viability of pancreatic cancer cells [14], as the study attributed these anti-proliferation effects to alterations in the redox state of cancer cells that Chl mediates [37] and leads to ROS formation [14]. However, lower concentrations showed no toxicity in cancer cells [10].

On the other hand, HepG2, MDA-MB-231, and HSF cell growth were unaffected by varied doses of the synthesized MSNs, up to 400 μg/mL, in the dark. In addition, numerous studies have shown that MSNs favor cell survival and act as a safe nanoparticle system [38]. The results showed a robust inhibitory impact at high concentrations when treating Chl-MSNs in the dark for the three cell lines. These findings could be attributed to the conjugate’s chlorophyll component; compared with Chl, Chl-MSNs are significantly more stable in a water-based solution, increasing the anticancer effect of chlorophyll [24].

**Fig. 3:**
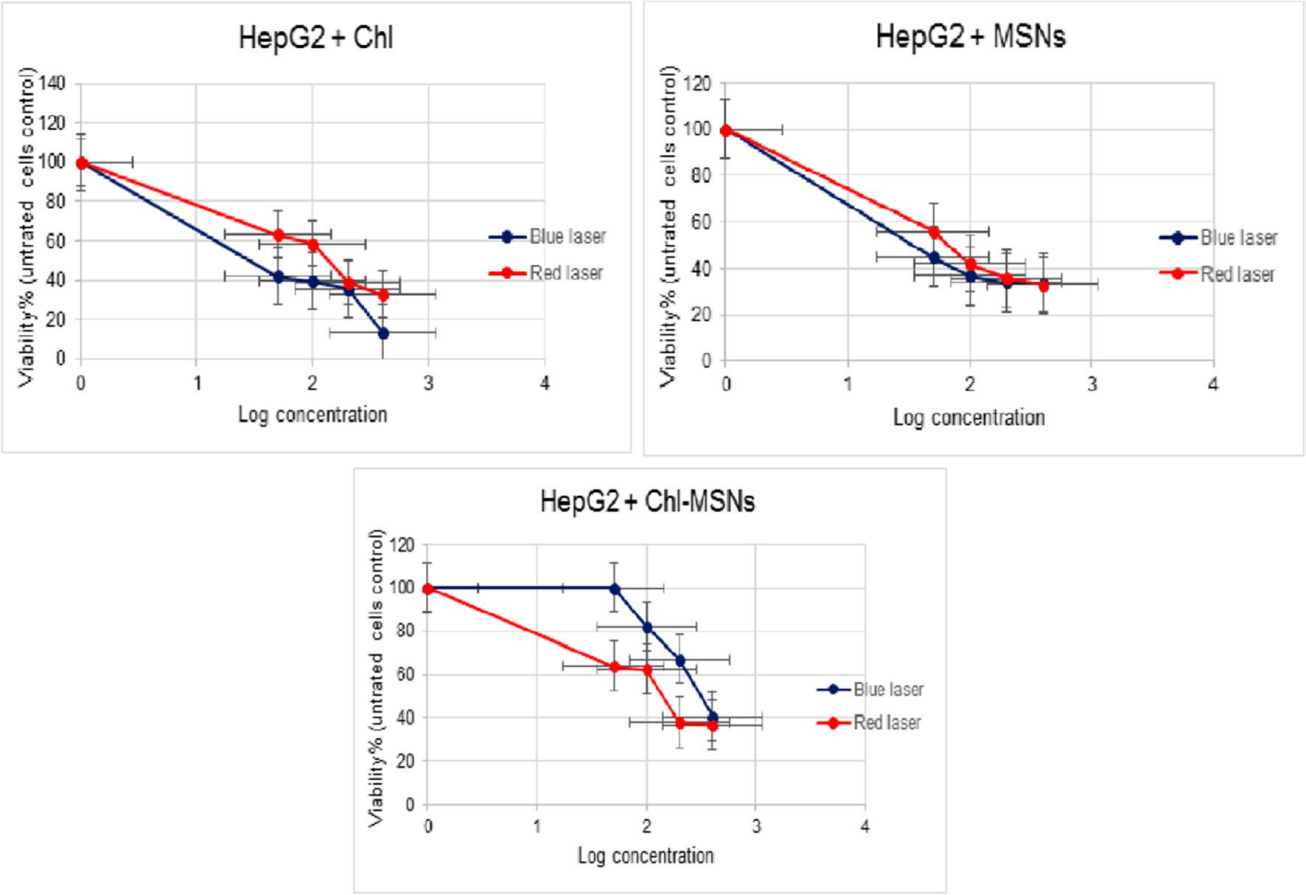
Effects of Chl, MSNs, and Chl-MSNs on HepG2 growth when exposed to blue and red lights. The results are presented as mean values with ± error bars

The PDT experiment results with red and blue lasers on HepG2 showed that when the cells were irradiated with 652 nm (light intensity of 20 mW/cm2) and 450–500 nm (light intensity of 100 mW/cm^2^) and in the presence of Chl, MSNs, and Chl-MSNs, respectively. The findings of the PDT with blue light were successful in inhibiting the growth of HepG2 cells other than red light in higher concentrations, and blue light with Chl only was more toxic than Chl-MSNs with IC50 values of 37.43 μg/mL and 310.9 μg/mL, respectively (Fig. 3). The blue radiation efficacy is due to a significant improvement in the anti-tumor effects of Chl in hepatic cancer cells by reducing viability via ROS production. In vitro irradiation with blue light increased its cytotoxicity against various tumor cells. This was demonstrated in different types of cancer. The combination of PSs that excites with blue light irradiation increases the cytotoxicity of PS to all epithelial liver tumor cells tested [39].

**Fig. 4:**
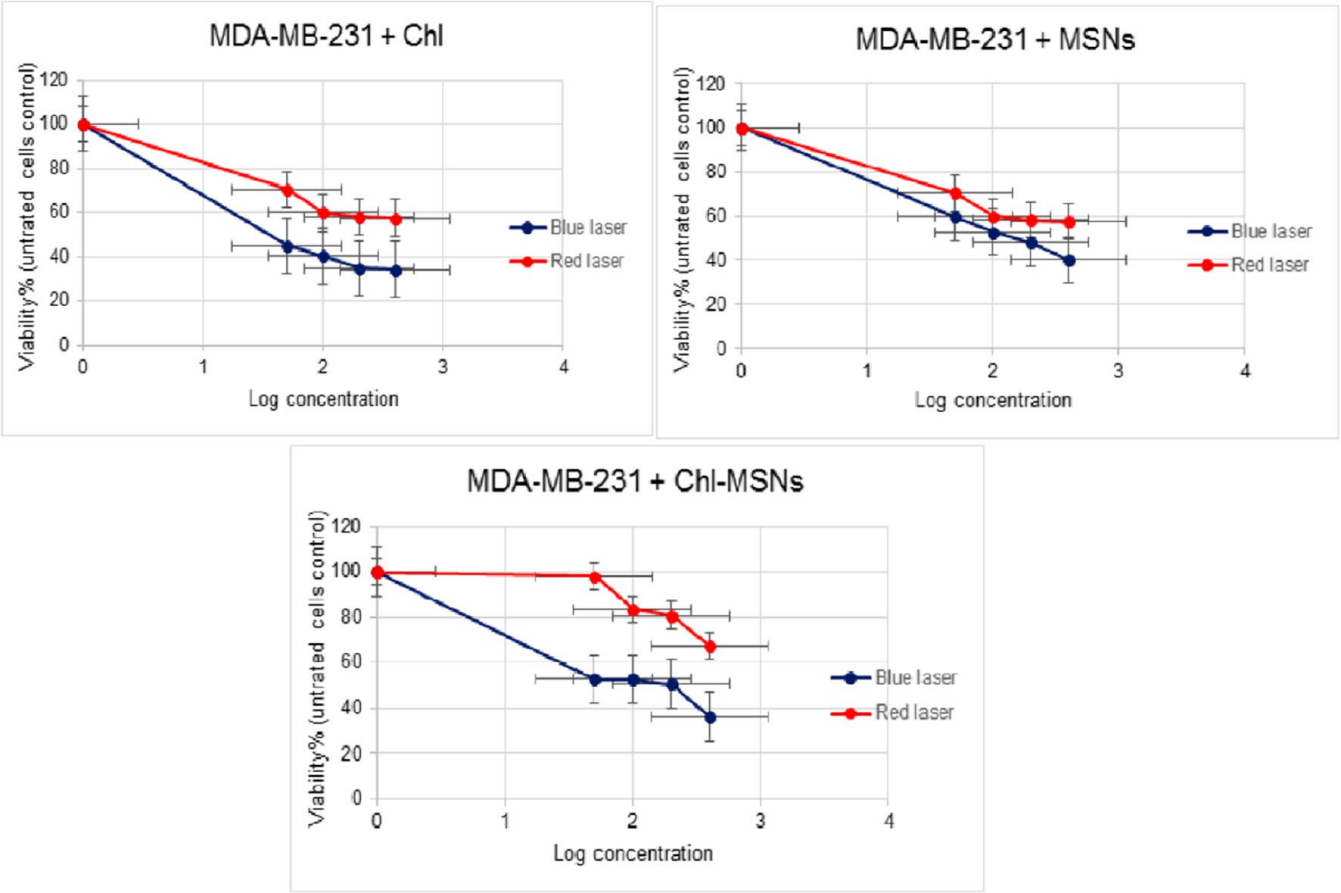
Effects of Chl, MSNs, and Chl-MSNs on MDA-MB-231 growth when exposed to blue and red lights. The results are presented as mean values with ± error bars

MDA-MB-231 cells resisted red radiation with Chl, MSNs, and Chl-MSNs. The toxicity was higher with Chl alone (IC50 = 18.89 μg/mL) than with Chl-MSNs (IC50 = 108.3 μg/mL), especially at high concentrations (Fig. 4). The efficiency of blue radiation may be explained by the fact that ROS generation is the primary outcome of PDT, which causes mitochondrial malfunction and cell death. When a PS is exposed to blue light, a significant amount of ROS is produced, which causes cancer cells to undergo apoptosis.

There is proof that ROS are early inducers of autophagy. These findings imply that PS excited by 450 nm, similar to Chl, may limit proliferation and trigger death in MDA-MB-231 cells by increasing intracellular ROS oxidative stress [40]. Breast cancer metastasis and recurrence can be effectively managed by PDT, as demonstrated by blue light. The resistance of MDA-MB-231 cells to red light radiation could be elucidated by the fact that a small quantity of PpIX in Chl enters mitochondria and decomposes into reactive oxygen species when exposed to light, which further protects cells from hydrogen peroxide damage and suppresses the production of ROS, as well as reducing heme production, which lowers the lethal effect of PDT and reduces the sensitivity of MDA-MB-231 cells to PDT [41].

**Fig. 5:**
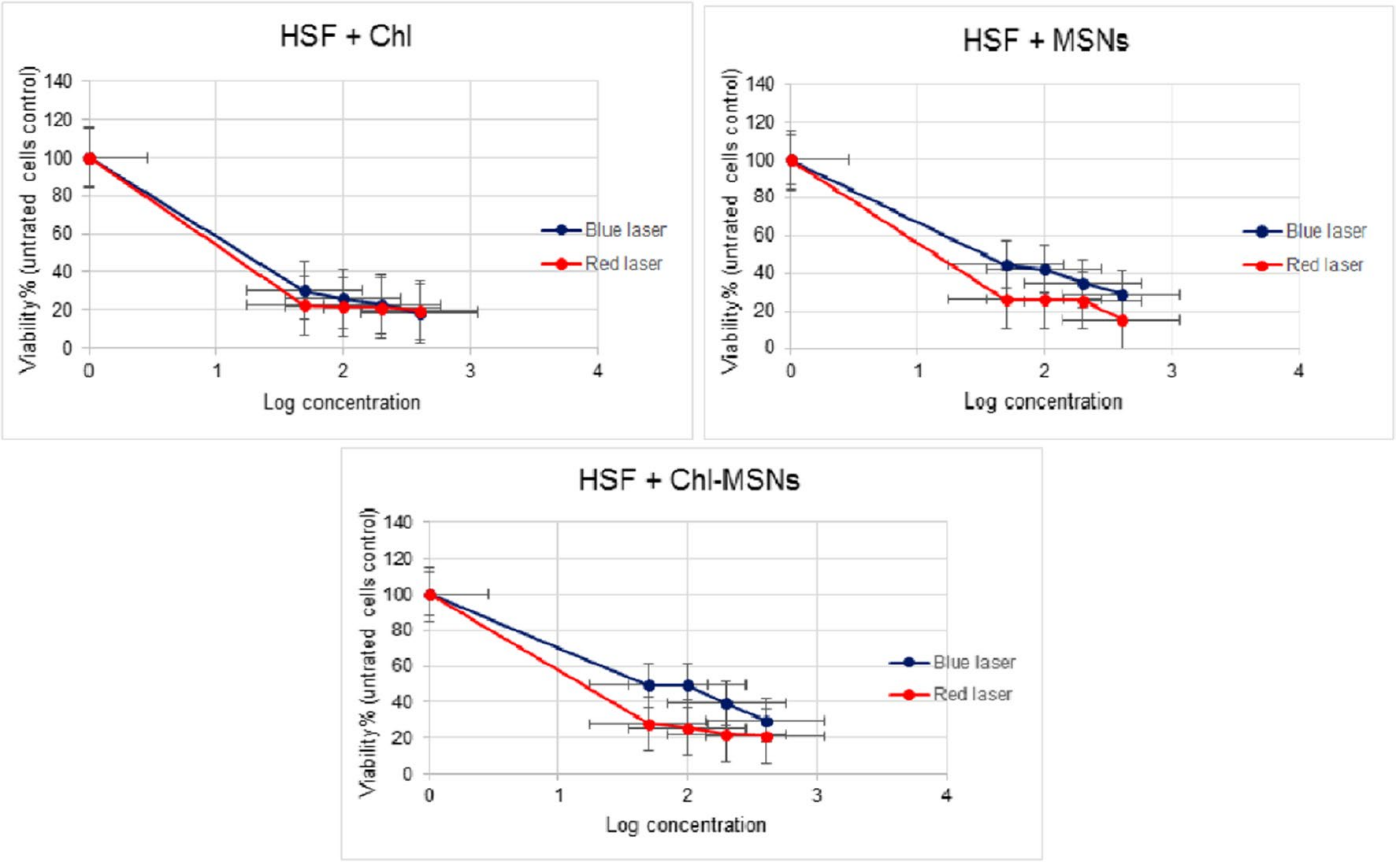
Effects of Chl, MSNs, and Chl-MSNs on HSF growth when exposed to blue and red lights. The results are presented as mean values with ± error bars

The results of PDT experiments using red and blue lasers on HSF cells showed that, at higher concentrations, in the presence of Chl, red light was more effective than blue light in suppressing the development of HSF cells (IC50 = 0.359 μg/mL) and Chl-MSNs (IC50 = 0.3226 μg/mL), respectively (Fig. 5). Blue light was slightly toxic to HSF cells compared with red light. In an earlier study, morphological analysis of standard skin specimens revealed that the structure of the tissue had been disturbed 15 days after PDT treatment, displaying inflammatory cell infiltration, responsive dermal fibroblasts, increased epidermal thickness, and a considerable decrease in collagen levels. Moreover, another sign of tissue remodeling is angiogenesis observed in normal skin cells. Therefore, modifications to the PDT protocol will be required to treat tumor cells, and increasing the number of sessions is anticipated to have a more substantial photodynamic effect [42].

MSNs did not exhibit any antiproliferative action toward any cancer cell lines when used at various concentrations in dark conditions, indicating that MSNs are an effective and safe nanoformulation for boosting the anticancer potential of Chl. However, some inhibition was observed at high concentrations when red and blue lights irradiated MSNs, particularly for the blue laser, which was significantly higher than the red laser in HepG2 and MDA-MB-231 cell lines. Except for HSF cells, the red laser was more potent with MSNs on the cells. This could be attributable to the focus on ROS-responsive therapeutic MSNs that release drugs in response to endogenous or external stimuli. Endogenous stimuli include pH, ROS, and temperature, whereas external triggers include X-ray and PDT [43].

Based on earlier studies that revealed Chl-MSN photo-efficiency, it was intended for the current work to demonstrate PDT’s combinational efficiency using red and blue lasers. Chl adsorption into the MSN pores results in a conjugate of Chl and MSNs with maximum absorption, making it relatively stable under illumination. This phenomenon may be caused by an interaction between two chlorophyll molecules, which results in a chlorophyll dimer and a tetrapyrrole ring transporting magnesium and the surfaces of the pores of MSNs. Following conjugation with MSNs, Chl molecules acquired excellent stability under light illumination, and the resulting Chl conjugate displayed high photosensitizing activity under prolonged illumination [24]. However, the research took a different path, showing that the efficacy of Chl with PDT is more potent than Chl with MSN conjugate. Nevertheless, Chl with MSNs can still be used as a safe formulation for removing tumor cells because it still has a mild anti-proliferation action because of Chl adsorption in MSNs.

## Conclusion

MSNs have effectively proved that they are unique nanoplatforms. Chl-MSN conjugate was advantageous for hydrophobic Chl, showing its higher stability in the aqueous environment and against light. PDT results with Chl, MSNs, and Chl-MSNs were better than dark conditions, showing that there is, indeed, a synergistic effect to limit tumor cell proliferation. Except for the Chl-MSN conjugate in the case of MDA-MB-231, blue laser is recommended over red laser with Chl and MSNs as a treatment for HepG2, MDA-MB-231, and HSF since red laser showed a weak toxic effect in the destruction of HepG2 and MDA-MB-231 cell lines in the presence Chl, MSNs, and Chl-MSNs. Moreover, red light exerted a high cytotoxic effect on HSF cells, which was shown to be unsafe for normal skin cells (HSF).

## Future work

Future studies aim to conduct in vivo experiments and prepare different concentrations of chlorophyll and Chl-MSN conjugate to evaluate the anticancer effects of photodynamic therapy, with further molecular studies required.

## Data Availability

All data needed to support the conclusions are included in this article. Additional data related to this paper can be requested from the author (fadyanajem@gstd.sci.cu.edu.eg or fadya2ad@gmail.com).

## List of abbreviations

°C: Degree Celsius
µL: Microliter
C2H5OCH2CH2OH: 2-Ethoxyethanol
CDNB: 1-Chloro-2, 4 dinitrobenzene
Chl: Chlorophyll
Chl-MSNs: Chlorophyll-loaded mesoporous silica nanoparticles
cm^2^: Square centimeter
CTAB: Cetyltrimethylammonium bromide
DA: Dalton
DLS: Dynamic light scattering
DMEM: Dulbecco’s modified Eagle medium
DMSO: Dimethyl sulfoxide
E.E.: Encapsulation efficiency
FBS: Fetal bovine serum
H_2_O_2_: Hydrogen peroxide
HPLC: High-performance liquid chromatography technique
IC_50_: Half maximal inhibitory concentration
J: Joule
mg: Milligram
mL: Milliliter
MSNs: Mesoporous silica nanoparticles
MTT: 3-(4,5 Dimethylthiazol-2-Yl) 2,5diphenyl tetrazolium bromide
mV: Millivolt
mW: Milliwatt
NaOH: Sodium hydroxide
NH4OH: Ammonium hydroxide
nm: Nanometer
NPs: Nanoparticles
OD: Optical densities
PBS: Phosphate buffered saline
PDI: Polydispersity index
PDT: Photodynamic therapy
PpIX: Protoporphyrin IX
PS: Photosensitizer
ROS: Reactive oxygen species
RPM: Revolutions per minute
S.M.E.: Standard error of the mean
SD: Standard deviation
TEM: Transmission electron microscope
TEOS: Tetraethyl orthosilicate
TEOS: Tetraethyl orthosilicate
UV-vis: Ultraviolet-visible
ZP: Zeta potential
*λ*: Wavelength
μg: Microgram
